# When Caring Becomes Suffering: Spirituality and Religiosity as Psychosocial Support for Cancer Caregivers—A Narrative Review

**DOI:** 10.3390/ijerph23040469

**Published:** 2026-04-07

**Authors:** Irineu Loturco

**Affiliations:** Department of Human Movement Science, Federal University of São Paulo, UNIFESP, São Paulo 11015-020, Brazil; irineu.loturco@terra.com.br

**Keywords:** mental health, coping strategies, emotional distress, depression, meaning-making

## Abstract

**Highlights:**

**Public health relevance—How does this work relate to a public health issue?**

- The rising global burden of cancer has expanded informal caregiving demands, exposing caregivers to sustained psychological distress, depression, and emotional overload, which represent underrecognized public health concerns.
- Caregiver mental health directly affects patient outcomes, healthcare utilization, and system sustainability, positioning psychosocial caregiver support as a critical component of comprehensive oncology care.

**Public health significance—Why is this work of significance to public health?**

- This review clarifies conceptual distinctions between religion, religiosity, and spirituality, reducing imprecision that can limit psychosocial research and enabling more rigorous investigation of caregiver mental health pathways.
- By synthesizing evidence across theoretical and empirical domains, it identifies potential mechanisms linking religiosity and spirituality to lower depressive symptomatology and improved psychological adjustment in cancer caregiving.

**Public health implications—What are the key implications or messages for practitioners, policy makers and/or researchers in public health?**

- Integrating spirituality- and religiosity-sensitive approaches into caregiver support programs can strengthen mental health strategies without replacing established psychological or psychiatric interventions.
- Future public health research should prioritize longitudinal, caregiver-specific, and culturally sensitive designs to clarify causal pathways and guide evidence-based policy in psycho-oncology and caregiver support services.

**Abstract:**

Cancer caregiving is increasingly recognized as a major psychosocial challenge, yet the mental health needs of caregivers remain insufficiently addressed in oncology research and practice. This narrative review examines the experiences of cancer caregivers within the context of rising cancer incidence and prolonged survival, conditions frequently accompanied by sustained psychological burden and anticipatory grief, with particular attention to depressive symptoms. Relevant qualitative and quantitative studies were identified through targeted searches of major databases (PubMed, Scopus, PsycINFO, and Google Scholar), including studies published up to January 2026. Study selection was guided by conceptual relevance and applied significance to the intersection between religiosity, spirituality, caregiving, and mental health outcomes. The reviewed literature highlights substantial psychological burden among caregivers, with depression affecting approximately 20–40% of cancer caregivers and identifies religiosity and spirituality as potentially supportive resources. Across studies, recurrent themes include meaning-making, hope maintenance, emotional regulation, moral orientation, and perceived social support as mechanisms through which these dimensions are associated with lower levels of depression and improved psychological adjustment. Evidence suggests that both religiosity, understood as the lived engagement with religious values, and spirituality, defined as a broader existential orientation toward meaning and purpose, contribute to coping in caregiving contexts; however, findings remain heterogeneous and largely based on cross-sectional analyses. Notable gaps persist, including limited caregiver-specific research, conceptual imprecision, and a lack of longitudinal designs. By integrating conceptual clarification with empirical synthesis, this review outlines potential psychological pathways linking religiosity and spirituality to caregiver mental health outcomes. In summary, religiosity and spirituality are considered adjunctive, non-exclusive resources that complement conventional psychological and psychiatric care within comprehensive models of caregiver support.

## 1. Introduction

Over recent decades, cancer incidence has increased steadily worldwide, affecting virtually all age groups and placing an ever-growing burden on societies and health systems [1,2]. This increase reflects demographic transitions such as population growth and aging, but also lifestyle changes, environmental exposures, and advances in diagnostic capacity, which together have contributed to a sustained rise in cancer diagnoses over successive decades [1,2]. Of particular concern is the increasing incidence and mortality observed among younger populations, a trend that challenges the long-standing perception of cancer as a disease predominantly associated with older age [3]. Despite continuous scientific advances in oncology—including improvements in early detection, surgical techniques, radiotherapy, chemotherapy, and targeted and immunotherapy approaches—these advances have not eliminated the clinical and psychosocial challenges arising from cancer diagnosis and treatment. Consequently, cancer remains one of the leading causes of death worldwide and is projected to surpass cardiovascular diseases as the primary cause of global mortality in the coming years [4]. This reflects the complexity of oncology care, as improvements in survival do not necessarily translate into proportional reductions in psychosocial burden.

Behind every oncology patient, there is an extensive, invisible network of support composed of family members, trusted friends, and healthcare professionals. Within this caregiving context, there is, almost invariably a primary caregiver, typically a close family member or someone with a strong emotional bond to the patient, such as a parent, spouse, sibling, or long-standing friend [5]. These caregivers take on some of the most emotionally demanding and psychologically challenging responsibilities. They witness physical decline, navigate difficult conversations, endure prolonged uncertainty, and remain constantly vigilant during long, sleepless nights marked by fear and anticipatory grief [6,7]. In everyday practice, this burden is rarely shared evenly. Caregivers rearrange their lives around the patient’s needs, assuming not only practical responsibilities but also substantial emotional and physical strain, frequently regulating their emotional responses [5]. They often feel compelled to suppress their suffering, adopting a posture of emotional control, even when the situation involves experiences of vulnerability, despair, and profound sadness [8]. This sustained emotional regulation, commonly carried out in silence and without recognition, represents a significant psychosocial cost, widely underestimated and largely neglected in clinical oncology [8].

Given these circumstances, it is possible to consider the magnitude of psychological overload experienced by these caregivers. Prolonged exposure to stress and uncertainty increases the risk of depressive symptoms, anxiety, and persistent psychological strain for these individuals [9,10]. Empirical evidence shows that caregivers of cancer patients frequently experience high levels of psychological burden, with estimates indicating that the prevalence of depressive symptoms ranges from approximately 20% to 40% across different caregiving contexts [8,11]. A defining characteristic of caregiving in oncology is the need to maintain a form of emotional duality: suppressing expressions of vulnerability to protect the patient from additional distress. Although psychiatry and psychology have made considerable progress in the treatment of psychological disorders, caregivers of oncology patients remain insufficiently supported [12,13]. This does not imply that conventional care is ineffective; rather, it remains incomplete when existential issues become central. Caring for someone who faces the prospect of suffering and death, particularly a loved one, exposes individuals to dimensions of experience that differ from those typically addressed in traditional psychotherapeutic methods, such as occupational stress, relational conflicts, or self-esteem concerns. Living with the daily awareness of finitude requires a distinct interpretive lens [14,15]. In many cases, there is still no clearly established solution for this form of suffering, nor a single intervention that consistently appears to provide lasting comfort. Alongside essential clinical care, adjunctive approaches that foster meaning, hope, and resilience can represent important complementary strategies [14,15].

Within this scenario, transcendent dimensions acquire particular relevance. Religion, religiosity, and spirituality emerge not only in informal conversations within hospitals, families, and caregiving settings, but also increasingly as topics of research. Despite the increasing global impact of cancer and the expanding body of literature focused on treatment innovation and patient-centered care, comparatively little attention has been devoted to the experiences and needs of cancer caregivers [5,13,16]. As a result, many interventions designed to address caregiver depression may not fully address the distinct emotional disturbances linked to anticipatory grief, illness uncertainty, and human and moral responsibility [12,17]—experiences rarely shared by other populations with depressive symptoms, even when clinical presentations appear similar. In this regard, religion, religiosity, and spirituality function as relevant adjunctive resources, supporting meaning-making and the interpretation of suffering (a process often described in the literature as “existential meaning-making” [15,18]), while sustaining hope and facilitating adaptive coping responses, helping individuals better understand, accept, and manage these experiences [19,20,21].

Therefore, the aims of this narrative review are to: (1) define and clearly distinguish between religion, religiosity, and spirituality; (2) identify and synthesize key studies examining how these dimensions support cancer caregivers experiencing depressive symptoms; and (3) propose practical pathways for integrating religiosity and spirituality into everyday caregiving contexts while highlighting directions for future research in this expanding field.

## 2. Materials and Methods

To address the aims of this narrative review, we prioritized studies that examined the role of religion, religiosity, and spirituality in the mental health and psychosocial well-being of caregivers of cancer patients. Rather than aiming for exhaustive coverage, study selection was guided by conceptual relevance, empirical contribution, and applied significance to caregiving contexts marked by psychological distress, existential suffering, and exposure to anticipatory loss. In this review, conceptual relevance was operationalized as the degree to which a study directly addressed the intersection between religiosity, spirituality, and the psychological or psychosocial experiences of cancer caregivers or closely related caregiving populations (e.g., palliative care contexts or family caregiving in severe illness) [10,22,23,24]. Applied significance referred to the extent to which the findings contributed to understanding clinically or psychosocially meaningful outcomes in caregiving settings, such as coping processes, emotional burden, depressive symptoms, meaning-making, or perceived social support resources [11,22,25]. Eligible studies included original empirical investigations and high-quality reviews published in peer-reviewed journals that addressed at least one of the following domains: (i) psychological distress, depression, anxiety, mental health disorders, or emotional burden among cancer caregivers; (ii) religious, spiritual, or existential coping processes in caregiving or palliative care settings; and (iii) associations between religiosity or spirituality and mental health outcomes in contexts of severe or chronic illness. Both qualitative and quantitative studies were considered, given the experiential and meaning-centered nature of the phenomena under investigation.

The literature search was conducted using established databases (i.e., PubMed, Scopus, Google Scholar, and PsycINFO), including studies published up to January 2026. The search strategy combined Boolean operators (e.g., AND, OR) and was adapted to the indexing specifications of each database. Search terms included combinations of keywords such as “cancer caregiver,” “oncology caregiving,” “religiosity,” “spirituality,” “religion,” “depression,” “mental health,” and “coping.” Only studies published in English were considered. Consistent with methodological recommendations for narrative literature reviews, the search strategy aimed to identify the most conceptually relevant and frequently cited studies addressing these constructs, rather than applying rigid inclusion or exclusion criteria typical of systematic reviews [22,25,26]. Initial screening was conducted based on titles and abstracts, followed by full-text evaluation when studies were deemed potentially relevant or conceptually significant. Study selection was informed not only by database retrieval but also by theoretical relevance and methodological robustness within the fields of psycho-oncology, palliative care, religiosity, spirituality, and health research. Given the narrative nature of this review, no formal risk-of-bias assessment or meta-analytic procedures were performed. Instead, emphasis was placed on critical interpretation, methodological consistency, and convergence of findings across studies. Editorials, opinion articles, and non-peer-reviewed materials were excluded, except for two widely cited seminal handbooks recognized as foundational references in their respective fields. This approach is consistent with established methodological descriptions of narrative reviews, which emphasize interpretive synthesis grounded in scholarly expertise rather than algorithmic study selection [22,25,26].

The conceptual structuring of the narrative review resulted in the selection of 83 references [5,6,7,8,9,10,11,12,13,14,15,16,17,18,19,20,21,22,23,24,25,26,27,28,29,30,31,32,33,34,35,36,37,38,39,40,41,42,43,44,45,46,47,48,49,50,51,52,53,54,55,56,57,58,59,60,61,62,63,64,65,66,67,68,69,70,71,72,73,74,75,76,77,78,79,80,81,82,83], of which 32 are organized into two tables: 15 references in Table 1 (Empirical Evidence in Cancer Caregivers and Related Contexts) and 17 references in Table 2 (Conceptual Foundations Informing the Narrative Review). The remaining references provide contextual and theoretical support for the study’s rationale and overall development. Table 1 and Table 2 are presented below. To facilitate critical discussion, the literature was organized into thematic sections addressing the following: (i) conceptual distinctions between religion, religiosity, and spirituality; (ii) the psychosocial and emotional burden of cancer caregiving; (iii) religiosity and spirituality as coping resources; (iv) potential mechanisms underlying their psychological effects on caregivers (e.g., improvements in meaning-making, hope, and social support); and (v) applied implications and future research directions. By organizing the review around these domains, this synthesis integrates empirical evidence with conceptual analysis to offer a coherent and clinically meaningful perspective on the role of religion, religiosity, and spirituality in supporting the mental health of cancer caregivers.

The studies presented in Table 1 focus on cancer caregivers and demonstrate that various forms of support and interventions—such as religiosity, spirituality, psychological counseling, psychiatric treatments, and social and financial assistance—are related to lower levels of depressive symptoms, better emotional adjustment, and improved coping capacity in cancer caregivers [5,6,13,27,28,29,30,31]. Furthermore, recent evidence suggests that spirituality may attenuate the negative psychological effects of prolonged caregiving by acting as a moderating factor in the relationship between caregiver burden and depression [29,32,33]. These findings are supported by research highlighting the importance of grief, meaning-making, and existential distress in caregivers’ experiences [17,27,30], as well as the need for multidimensional measures to better capture the complexity of caregiver burden and psychological responses [12,29,31]. Overall, these results support the view that religiosity and spirituality function as multidimensional resources influencing caregivers’ mental health through interconnected cognitive, emotional, social, and existential pathways. Table 2 presents studies that provide the conceptual foundation for this review, including research involving other populations as well as methodological analyses of caregiving in chronic illness contexts. These studies address both religious and spiritual dimensions, in addition to the broader challenges faced by family and professional caregivers, such as stress management, coping strategies, and the impact of caregiving on personal well-being.

## 3. Conceptual Distinctions Between Religion, Religiosity, and Spirituality

Religion is defined as an organized system of beliefs, doctrines, practices, rituals, and moral norms oriented toward the sacred or transcendent, commonly shared within a given community [20,34]. As a collective and socially regulated phenomenon, religion is expressed through formal institutions, normative texts, and codified practices that structure belief and behavior within a specific tradition [35,36]. From a sociological perspective, religion provides a symbolic framework that may shape values, worldviews, and moral orientations at the group level [35]. In health-related research, religion is therefore understood primarily as an external and institutional system rather than as an individual psychological or social attribute [19,20]. Religiosity, while closely related to religion, refers to the degree of individual involvement, commitment, and engagement with a religious tradition [23]. It reflects how religion is personally lived, internalized, and practiced, rather than the institutional structure itself.

Religiosity may be expressed through public behaviors (e.g., attendance at religious services) as well as private practices and through the subjective importance attributed to religious beliefs in daily life. More broadly, religiosity can be understood as a system of established beliefs, doctrines, rituals, and ethical norms that guide conceptions of a “good life” [23,37]. Crucially, individuals who share the same religious affiliation differ substantially in both the level and expression of religiosity, underscoring the distinction between belonging to a religious tradition and actively embodying it at the individual level [19,23,34]. Hence, the ways in which individuals embody their faith occasionally diverge from the standardized doctrines of a formal religion. In certain contexts, isolated actions performed by specific groups may influence public perceptions of that religious tradition [38,39].

Lastly, spirituality represents a more comprehensive and individualized construct, typically defined as the personal search for meaning, purpose, and connection with what is perceived as sacred, transcendent, and significant. Unlike religion, spirituality is not necessarily tied to institutional affiliation, conventional doctrine, or communal rituals and can be expressed both within and outside religious environments. Spirituality emphasizes subjective experience, existential reflection, and inner resources, often focusing on meaning-making, coherence, and connection in the face of life adversity and daily challenges [32,40,41]. Although these constructs partially overlap, their theoretical distinction is considered important, particularly in health, psychosocial, and clinical research. While religion refers to an institutional system of beliefs and practices, religiosity reflects the personal engagement through which faith and compassion are expressed in everyday life [23,42,43]. In turn, spirituality refers to a more encompassing existential orientation that may or may not involve religious traditions [23,44,45].

Empirical evidence generally indicates that psychological and health-related outcomes tend to be more strongly connected to high levels of religiosity and spirituality than to religious affiliation alone, highlighting the importance of differentiating these dimensions when examining resilience and mental health in contexts of illness and caregiving [34,46,47]. A lack of clear distinction between these constructs may result in conceptual imprecision that obscures the mechanisms linking transcendent resources to coping processes and mental health outcomes in caregiving contexts, such as how different aspects of spirituality uniquely contribute to resilience in caregivers facing chronic illness challenges. These conceptual distinctions also carry important implications for measurement in caregiver research. Studies that rely solely on religious affiliation or single-item indicators fail to capture the multidimensional and experiential nature of religiosity and spirituality, potentially leading to inconsistent or attenuated associations with mental health outcomes. In contrast, approaches that differentiate between organizational, nonorganizational, and intrinsic dimensions of religiosity, as well as broader indicators of spiritual well-being and meaning, may provide a more accurate and nuanced understanding of how these constructs relate to depressive symptoms, emotional regulation, and coping in cancer caregivers. Therefore, careful alignment between conceptual definitions and measurement strategies is essential to strengthen empirical investigations and improve the interpretability of findings in this field [23,24,48].

## 4. The Common Premises of Religious Traditions

Despite their doctrinal, cultural, and ritual differences, religious traditions are often described as sharing core moral premises oriented toward the promotion of common human values and the affirmation of good over evil [49]. Through ethical teachings, sacred narratives, and ritual practices—even within traditions with less formalized rituals—religions usually articulate normative structures that emphasize compassion, justice, forgiveness, and responsibility toward others. In periods of intense distress, uncertainty, and suffering, such as those experienced by caregivers of patients with cancer, these shared foundations may function as accessible sources of emotional support, meaning-making, and psychological stability, especially when deeply embedded in personal and collective experiences [48,49,50,51].

Across philosophical, theological, and psychological perspectives, there is growing recognition that the value of religion is more closely related to ethical conduct in everyday life than to ritual observance alone [48,52]. Classical authors such as Augustine and Aquinas emphasized that an authentic religious life is expressed through charity, compassion, and forgiveness, while Kant similarly highlighted moral action grounded in duty and respect for others as central to religious experience [51,53,54]. In modern psychology, this distinction is reflected in the differentiation between extrinsic religiosity (instrumental or socially driven) and intrinsic religiosity (internalized and consistently expressed in behavior) [52,55]. Within this framework, the alignment of actions with ethical principles such as love, solidarity, and concern for others emerges as a central dimension of religious expression [56,57].

Religious practices or interpretations that promote exclusion, hostility, or division may contradict these principles and may adversely affect emotional regulation and psychological well-being. In caregiving contexts, where relational stability, empathy, and emotional balance influence mental health, this distinction holds particular relevance [5,58]. Consequently, differentiating religion as an institutional system from religiosity as a lived, ethically grounded orientation—while also differentiating both constructs from spirituality—may enhance the comprehension of the psychosocial mechanisms through which these dimensions influence caregiver health. Ultimately, everyday attitudes and actions grounded in ethical values, rather than ritual practices alone, appear to represent more meaningful and psychologically supportive expressions of religious life in this population.

## 5. Religiosity as a Supportive Resource for Mental Health in Cancer Caregivers

As mentioned above, a more detailed examination of religiosity is now warranted, especially in relation to mental health among caregivers of cancer patients. In general terms, religiosity is defined as the degree to which individuals internalize, value, and actively live out their beliefs, moral principles, and practices grounded in a religious tradition in their lives [19,23]. Unlike religion as an institutional system, religiosity reflects a subjective, experiential, and ethically grounded orientation that shapes meaning-making, a sense of purpose, coping strategies, and interpersonal behavior [19,23]. Hence, religiosity extends beyond ritual observance or formal affiliation, encompassing dimensions such as personal faith, moral commitment, informal forms of prayer, trust in a transcendent order, and the integration of religious values into everyday decision-making processes [19,59,60]. These elements become relevant in situations marked by chronic stress, uncertainty, sadness, and emotional burden, conditions that characterize the caregiving experience in oncology. As mentioned earlier, depression affects 20–40% of cancer caregivers, depending on the stage of the disease and the assessment instruments used, highlighting the magnitude of the psychological burden experienced in this population [8,11]. In such settings, religiosity serves as a source of emotional regulation and resilience, thus acting as a relevant adjunctive resource in supporting the management and mitigation of psychological distress, with implications for depressive outcomes.

A substantial body of research across diverse clinical populations supports an association between religiosity and improved mental health outcomes [58]. Investigations involving individuals with chronic illness, major depression, and trauma-related disorders—including veterans exposed to combat [58,61]—have consistently shown that higher levels of religiosity are accompanied by lower depressive symptomatology, reduced anxiety, greater psychological resilience, and enhanced coping capacity [58,61,62]. In trauma-exposed populations, religious coping has been linked to the reconstruction of meaning and life purpose, reduced hopelessness, and improved emotional adjustment, with stronger effects observed when religious beliefs are internalized rather than used instrumentally [58,61,62]. Despite this extensive literature, few studies have specifically examined the role of religiosity in the mental health of caregivers of cancer patients [48]. Caregiving in oncology is characterized by a distinct set of stressors that differentiates this population from other caregiving and clinical contexts [5,7]. Unlike many chronic conditions, cancer caregiving frequently involves the simultaneous coexistence of hope for recovery and the anticipation of potential loss, creating a persistent state of emotional duality [6,7]. Caregivers are usually required to regulate their distress while maintaining a supportive and optimistic presence for the patient, which may intensify emotional suppression and internal conflict [5]. In this context, the utilization of general models of religious coping assumes unique characteristics, as meaning-making processes are aimed not only at stress management but also at the integration of uncertainty, finitude, and anticipatory grief into a cohesive psychological framework [18,24].

This represents a notable gap, given the unique set of stressors faced by this population, including anticipatory loss, prolonged exposure to suffering, and the frequent need to suppress their own emotional states to avoid inducing guilt or additional psychological distress in patients [5]. Although the evidence is limited, it suggests a positive relationship between religiosity and the mental health of oncologic caregivers [63]. Two previous investigations (i.e., one prospective longitudinal observational study and one cross-sectional observational study) have shown that higher levels of religiosity among cancer caregivers are related to lower levels of depressive symptomatology, greater emotional well-being, and improved perceived coping capacity [28,64]. In these studies, caregivers who reported more consistent religious engagement demonstrated greater acceptance of the caregiving role, reduced feelings of helplessness, and a more coherent process of meaning-making through which to interpret, accept, and cope with emotional challenges and loss.

These benefits do not appear to stem solely from the social support provided by religious communities but also from internal processes such as existential orientation, hope maintenance, moral orientation, and a perceived connection with a transcendent and hope-affirming source of support [48]. Religiosity may assist caregivers in reinterpreting suffering, which is commonly perceived in religious traditions as a means of learning that promotes personal development and a reassessment of fundamental human values. It also sustains motivation in the absence of control over outcomes and supports emotional regulation when immediate or desired resolutions are not possible [24,48]. These combined effects are consistent with theoretical models of religious coping, which emphasize the role of faith-based meaning systems in managing uncontrollable stressors that contribute to elevated levels of depression [24,40,65]. Nevertheless, it is essential to acknowledge that this field of research remains largely underdeveloped and that observed correlations do not necessarily imply causation. The majority of existing studies are cross-sectional, employ various measures of religiosity, and rarely focus exclusively on cancer caregivers [8,11].

Despite these limitations, the convergence of evidence indicates that caregivers with higher levels of religiosity show better mental health, particularly in terms of depressive symptoms and emotional distress. Given the projected global increase in cancer incidence and the growing reliance on informal and often unprepared caregiving [2,13], this line of research would benefit from further expansion. Accordingly, future research should prioritize longitudinal designs with caregiver-specific outcomes and culturally sensitive measures to better capture the complex and heterogeneous ways in which religiosity supports psychological well-being in oncology caregiving contexts.

## 6. Spirituality as a Supportive Resource for Mental Health in Cancer Caregivers

Despite recurrent misunderstandings and imprecise interpretations, religiosity and spirituality, although interconnected, do not represent the same constructs. Religiosity is understood as the degree to which individuals adhere to, internalize, and practice beliefs and rituals within an organized tradition, whereas spirituality refers to a broader, more subjective orientation toward meaning, purpose, connectedness, and transcendence in life [19,23]. Unlike religiosity, spirituality can be described as an experiential dimension oriented toward meaning-making, inner integration, and values that guide behavior and social conduct, irrespective of formal religious affiliation, especially in contexts of psychological distress [18,21,66]. Under this perspective, individuals who do not identify as religious may nonetheless have their lives shaped by spiritual principles expressed through compassion, charity, and altruism. However, the present review adopts a more focused perspective, examining spirituality primarily within a conceptual scheme interrelated with religion and religiosity, given the substantial overlap among these domains and their shared relevance in caregiving contexts [19,52].

Spirituality, in its essence, is increasingly recognized as a potentially valuable factor in mitigating emotional burden among practitioners, caregivers, and patients across a wide range of chronic and life-threatening conditions [19,67]. Research indicates that spiritual orientation acts as an auxiliary coping mechanism, assisting individuals in managing feelings of helplessness and social isolation while providing a psychological refuge for those who must endure suffering in silence [68]. Studies involving caregivers of patients with neurodegenerative diseases, cardiovascular conditions, and advanced chronic illnesses show that spirituality is linked to reduced emotional exhaustion, fewer depressive symptoms, and enhanced emotional balance [33]. Similar findings have been reported among healthcare professionals, including nurses and physicians, for whom spiritual aspects are related to a reduced psychological impact of sustained emotional pressure, moral distress, and occupational burnout [69,70,71].

More recent investigations in oncology caregiving contexts further support these observations, indicating that spirituality also plays a protective role in grief-related processes and emotional adjustment following cancer-related loss, reinforcing its relevance across different stages of the caregiving trajectory [27]. Despite these observations, the literature remains limited regarding the actual role of spirituality in supporting the mental health of cancer caregivers, specifically with regard to depressive symptoms and psychological maladjustment, indicating a persistent gap in this field. This limitation is especially relevant given the specific emotional and existential demands of cancer caregiving, which involve continuous exposure to suffering, uncertainty regarding disease progression, and anticipation of loss [5,6,7,17]. As a result, spirituality in oncology caregiving functions not only as a general coping resource but also as a framework for processing finitude, sustaining hope under uncertainty, and reconciling conflicting emotional experiences [18,24,29]. Thus, its effects appear to be context-dependent and closely connected to caregiver experiences rather than fully explained by general theoretical models [24,29,33].

Emerging evidence, although scarce, suggests that spirituality appears to play a meaningful role in sustaining mental health among caregivers of cancer patients [8,72]. Available studies indicate that higher levels of spiritual well-being are associated with lower levels of depressive symptoms, greater emotional acceptance, and improved psychological adjustment in oncology and other chronic or degenerative caregiving contexts [64,65,73]. Additionally, spirituality seems to reduce the negative effects of caregiver stress on depression, reinforcing its role as a relevant psychological resource in this population [29]. These relationships appear to be partly explained by spirituality’s capacity to reinforce hope, promote psychological anchoring, and support adaptive reinterpretations of suffering and loss [24,51]. Moreover, systematic evidence concerning grief assessment in oncology settings indicates that emotional and existential dimensions—frequently aligned with spiritual constructs—are central to understanding caregiver distress, reinforcing the necessity to incorporate spirituality into comprehensive conceptual and measurement frameworks [30]. This relevance extends beyond formal religious identification, as spiritual constructs can also benefit individuals who identify as agnostic or atheist [34,74]. Such wide applicability supports the potential of spirituality as an adjunctive resource that works alongside established psychiatric and psychological interventions used to address mental disorders associated with modern stressors.

In fact, spirituality does not necessarily depend on formal belief systems; rather, it may be expressed through everyday attitudes, ethical commitments, and meaning-oriented reflection [60,66,68]. When integrated into caregivers’ lived experiences, spirituality contributes to the development of renewed purpose, facilitates acceptance, and supports adaptive responses to prolonged stress and loss [15,24,48,65]. The search for meaning and purpose has been consistently identified as a protective factor and as a complementary component in the management of depressive symptoms [15,24]. Considering the high prevalence of depressive symptoms among caregivers of cancer patients—with studies suggesting that roughly one-third of caregivers report clinically significant symptom levels during the caregiving trajectory [8,11]—and acknowledging that the current evidence base remains limited, available findings suggest relationships between spiritual engagement and mental health indicators in this population. In this sense, distinguishing spirituality from more rigid or institutional definitions of religion and religiosity may enhance its applicability as a supportive resource for managing depression and other affective conditions commonly observed among cancer caregivers.

## 7. Mechanisms and Pathways Linking Religiosity and Spirituality to Mental Health in Cancer Caregivers

Although the scientific literature directly examining religiosity and spirituality in cancer caregivers remains limited, converging theoretical models and findings from related populations allow for the identification of several potential mechanisms through which these dimensions influence caregivers’ mental health outcomes [75]. Empirical studies and systematic reviews reinforce the need to integrate these mechanisms with caregiver-specific outcomes and measurement approaches, particularly in oncology contexts where burden, grief, and psychological distress are multidimensional constructs [30,31].

### 7.1. Cognitive and Emotional Pathways

Central to these pathways is the role of religiosity and spirituality in shaping cognitive appraisal and emotional regulation processes under conditions of sustained stress, persistent sadness, and suffering [24,76]. Models of religiosity and spiritual coping emphasize that the internalization of religious beliefs and meaningful practices encourages adaptive interpretations and cognitive functioning, enhances psychological resilience in response to uncontrollable stressors, and supports greater acceptance of adversity [48]. These processes are consistent with meta-analytic evidence indicating that caregiver burden is strongly associated with anxiety and psychological distress, particularly when maladaptive cognitive patterns are not effectively regulated [77]. In caregiving contexts marked by uncertainty, high levels of sadness, anticipatory distress, and emotional strain, these processes can attenuate maladaptive cognitive patterns such as persistent negative thinking, which are prevalent across common psychological disorders and depressive conditions [78,79].

By offering alternative regulatory strategies and sources of emotional support that situate suffering within broader existential or moral narratives, religiosity and spirituality contribute to more adaptive responses to prolonged caregiving demands characterized by substantial psychological pressure [24,68]. The need to navigate emotional duality and anticipatory grief further shapes these cognitive and emotional processes [5,6,7,15]. Caregivers commonly oscillate between maintaining hope and preparing for potential loss, which may amplify cognitive load and emotional strain [6,7,27]. Hence, the regulatory role of religiosity and spirituality in this scenario extends beyond general stress adaptation, encompassing the integration of conflicting emotional states and the construction of meaning in the presence of sustained uncertainty and existential adversity [18,24,73].

### 7.2. Hope- and Purpose-Related Pathways

A second set of pathways relates to the capacity of religiosity and spirituality to reinforce hope, purpose, and psychological stability during periods of chronic suffering and clinically significant depressive symptomatology [48,62]. The reconstruction of a sense of purpose has been consistently identified as a protective factor against depression and emotional exhaustion in populations exposed to long-term stress, adversity, and the possibility of loss [15,18,19]. In oncology caregiving, maintaining hope is inherently complex, as it must coexist with an awareness of potential decline and loss [5,6,13,16]. This dynamic sets it apart from other caregiving situations, where clinical courses are more predictable, such as in cases of chronic illness with established treatment protocols [7,15]. As a result, caregivers are required to navigate ongoing uncertainty about their loved ones’ health [5,6,27]. In this setting, meaning- and purpose-related processes function not only as protective factors but also as ways of integrating hope with uncertainty, helping caregivers remain engaged despite emotional ambiguity and an almost uncontrollable fear of losing a loved one [15,18,24,80]. Recent oncology-specific evidence further suggests that spiritual and existential resources play a central role in grief processing and meaning reconstruction among caregivers, particularly in the context of anticipatory or post-loss adaptation [27,30].

In cancer caregiving, where control over outcomes is always limited and emotional rewards are usually delayed or ambiguous, spiritual perspectives support motivation by sustaining long-term perspectives and reinforcing the perceived value of the caregiving role [73]. Additionally, the moderating role of spirituality in the relationship between caregiver burden and depression suggests that hope- and meaning-related practices may buffer the negative psychological impact of sustained caregiving demands [29]. These methods have been connected to less emotional withdrawal, more persistence in caregiving tasks, greater resilience, and increased hope over time. Such benefits do not depend exclusively on formal religious adherence; rather, they are derived from deeper spiritual commitments based on ethical values, compassion, and human responsibility toward others [81]. Thus, religiosity and spirituality are described as stabilizing internal resources that help caregivers maintain balance and continuity of identity amid ongoing, demanding, and emotionally taxing caregiving demands [24,29,48,50].

### 7.3. Social and Clinical Pathways

Additional pathways are mediated through indirect clinical mechanisms that influence social support, ethical orientation, and interaction with healthcare resources [19,82]. Religiosity and spirituality are associated with increased perceived social support by fostering a sense of belonging, moral validation, and shared meaning within communities, which helps counteract social isolation and emotional loneliness frequently reported by caregivers [64,82]. In addition, the literature emphasizes that assessing caregiver burden necessitates multidimensional measurement approaches, as various instruments capture distinct emotional, social, and existential domains of caregiving-related stress [31]. These dimensions are essential for understanding the full impact of caregiving on mental health and well-being. Furthermore, spiritual orientations and religiosity validate expressions of vulnerability, promote help-seeking behaviors, and enhance access to psychological and psychiatric support when required [19,48,64]. Collectively, these factors facilitate access to mental health interventions, while promoting adaptive functioning and sustained engagement in treatment processes over time [19,80].

However, it is important to recognize that not all forms of religious or spiritual coping are necessarily adaptive. An increasing body of literature has identified maladaptive or negative religious coping patterns, such as spiritual struggle, feelings of abandonment or punishment by a higher power, rigid or fatalistic belief systems, and the use of religion to justify avoidance or emotional suppression [58,65,83]. These responses may exacerbate psychological distress, heighten vulnerability to depressive symptoms, and hinder adaptive coping, especially in contexts marked by extended uncertainty and emotional strain, such as cancer caregiving.

The effects of religiosity and spirituality on mental health are not uniformly positive; rather, they depend on the nature, interpretation, and integration of these beliefs and practices within the individual’s coping framework. Taken together, these domains suggest that religiosity and spirituality play a role in shaping caregiver mental health and its expression through interconnected cognitive, emotional, social, and existential pathways [48]. Although direct causal evidence in oncology caregiving is limited, the alignment of findings across theoretical models and related populations offers a reliable basis for understanding how these dimensions may alleviate depressive symptoms commonly experienced by cancer caregivers and contribute to improvements in their emotional well-being and mental health. This convergence supports the relevance of religiosity and spirituality as adjunctive resources within comprehensive support models for cancer caregivers [19,24].

## 8. Limitations

This review presents several limitations that should be acknowledged. First, the available evidence remains limited and is predominantly composed of cross-sectional studies, which restricts causal inferences regarding the relationships between religiosity, spirituality, and mental health outcomes in cancer caregivers. Second, the diversity in conceptual definitions and measurement methodologies for religiosity and spirituality leads to inconsistencies among studies, thus restricting the comparability of results. Third, although this review prioritized conceptual relevance and applied significance, its narrative design does not follow a systematic protocol, which may introduce selection bias and limit reproducibility. Additionally, relatively few studies have specifically focused on cancer caregivers, with many findings derived from related populations, potentially reducing the specificity of the conclusions. Lastly, cultural variability in the expression of religiosity and spirituality can influence both coping processes and mental health outcomes, thereby limiting the generalizability of the findings across different contexts. Despite these limitations, to the best of our knowledge, this review is the first to simultaneously delineate the distinctions between religion, religiosity, and spirituality, thereby offering a novel perspective to support individuals living in contexts profoundly shaped by silent and enduring suffering.

## 9. Conclusions

This narrative review synthesizes conceptual and empirical evidence suggesting that religiosity and spirituality may function as complementary resources in supporting the mental well-being of cancer caregivers. Although the current evidence remains limited and predominantly cross-sectional, converging findings indicate potential benefits related to meaning-making, emotional regulation, hope maintenance, and perceived social connectedness. Given the narrative nature of this review and the reliance on conceptually relevant evidence, including findings derived from adjacent populations and theoretical models, the present conclusions should be interpreted as propositional in nature and intended to provide a conceptual foundation to guide future systematic reviews and longitudinal studies in this field. These dimensions should not be interpreted as alternatives to established psychological or psychiatric interventions but rather as adjunctive components within comprehensive caregiver support models [13]. Future research should prioritize longitudinal and culturally sensitive investigations to clarify causal pathways and optimize clinical integration. The findings of this review also highlight that the relevance of religiosity and spirituality extends beyond individual coping, encompassing broader relational, ethical, and social dimensions that shape the caregiving experience. By influencing how caregivers interpret suffering, sustain motivation, and maintain emotional balance under prolonged stress, these constructs can foster more adaptive psychological trajectories over time. Accordingly, religiosity and spirituality appear to operate as integrative structures that connect cognitive, emotional, and existential processes in contexts characterized by uncertainty, loss, and limited control. From a clinical and public health perspective, acknowledging these dimensions promotes the development of more tailored care models. Integrating religiosity- and spirituality-sensitive approaches into caregiver support programs does not require replacing evidence-based interventions but instead involves their contextual enrichment, particularly in settings where existential concerns are insufficiently addressed by conventional approaches. Such integration may enhance engagement with care, adherence to interventions, and overall psychological adjustment among caregivers. However, the heterogeneity of constructs, measures, and study designs in the current literature warrants caution. The lack of caregiver studies specifically addressing religious or spiritual dimensions, combined with the limited use of longitudinal methodologies and variability in conceptual definitions, restricts the robustness of causal inferences. Future research should emphasize theoretically informed and methodologically robust designs, incorporating longitudinal and mixed-method approaches, to more effectively elucidate the dynamic and context-sensitive characteristics of religiosity and spirituality within caregiving trajectories. Furthermore, culturally sensitive frameworks are essential, as expressions of religiosity and spirituality vary substantially across populations and influence both coping strategies and mental health outcomes. Advancing this field will require not only improved measurement and conceptual clarity but also deeper integration of interdisciplinary perspectives spanning psycho-oncology, public health, psychology of religion, and behavioral sciences. When appropriately contextualized and integrated, these dimensions may contribute to more comprehensive, responsive, and human-centered approaches to supporting caregiver well-being.

## Figures and Tables

**Table 1:** Empirical Evidence in Cancer Caregivers and Related Caregiving Contexts.

Author (Year)	Study Type	Population	Central Focus	Main Contribution
Northouse et al. [5]	Meta-analysis	Cancer caregivers	Interventions	Interventions reduce burden and distress
Given et al. [6]	Narrative review	Advanced cancer caregivers	Family support	Highlights of family support in advanced cancer care
Stenberg et al. [7]	Review	Cancer caregivers	Impact of caregiving	Identifies multidimensional burden (emotional and social)
Applebaum & Breitbart [8]	Systematic review	Cancer caregivers	Psychological distress	High prevalence of depression and unmet needs
Schulz & Sherwood [10]	Review	Family caregivers	Physical and mental health	Caregiving associated with adverse health outcomes
Geng et al. [11]	Meta-analysis	Cancer caregivers	Depression prevalence	Elevated depressive symptoms and unmet needs
Adelman et al. [12]	Clinical review	Informal caregivers	Caregiver burden	Defines caregiver burden and clinical implications
Kent et al. [13]	Review	Informal cancer caregivers	Research priorities	Identifies clinical priorities in cancer caregiving
Karacan et al. [27]	Observational study	Bereaved cancer caregivers	Grief and well-being relationship	Family caregivers after cancer loss
Kim et al. [28]	Empirical study	Survivor–caregiver dyads	Spiritual well-being	Higher spiritual well-being associated with better * QoL
La et al. [29]	Observational study	Cancer caregivers	Spirituality moderating depression	Spirituality buffers burden–depression link
Mattson et al. [30]	Systematic review	Cancer caregivers	Grief assessment via psychometrics	Evaluates grief measures
Zhong et al. [31]	Review	Informal cancer caregivers	Caregiver burden assessment tools	Evaluates burden measures
Kim & Schulz [72]	Empirical study	Cancer caregivers	Caregiver strain	High psychological burden compared to other groups
Pearce et al. [73]	Empirical study	Terminal cancer caregivers	Religious coping	Positive religious coping linked to better adjustment

**Note:** * QoL = Quality of Life.

**Table 2:** Conceptual and Theoretical Foundations Informing the Narrative Review.

Author (Year)	Study Type	Core Construct	Relevance & Contribution to Present Review
Frankl [18]	Handbook	Meaning	Existential meaning
Koenig [19]	Review	Religion & health	Mental health associations
Koenig et al. [20]	Handbook	Religion & health	Clinical synthesis
Hill & Pargament [23]	Conceptual article	Religion vs. spirituality	Clarifies construct differentiation
Park [24]	Review	Meaning-making	Stress adjustment
Pargament [40]	Conceptual article	Religion vs. spirituality	Theoretical distinction
Zinnbauer et al. [41]	Conceptual article	Spirituality	Foundational conceptual framework
Allport & Ross [52]	Conceptual article	Intrinsic/extrinsic religiosity	Motivational model
Pargament et al. [58]	Methodological study	* RCOPE	Positive/negative coping
Smith et al. [62]	Meta-analysis	Religiosity	↓ Depression
Pargament et al. [65]	Conceptual article	Religious coping	Stress coping model
Weber & Pargament [75]	Review	Neurocognitive mechanisms	Cognitive/social pathways
Folkman [76]	Conceptual article	Stress appraisal	Coping process
Del-Pino-Casado et al. [77]	Meta-analysis	Caregiver burden and anxiety	Supports caregiver burden–mental health link
Nolen-Hoeksema et al. [78]	Conceptual article	Rumination	Depression mechanism
George et al. [82]	Review	Social support	Religion–health pathway
Lazarus & Folkman [83]	Handbook	Stress & coping	Transactional stress model

**Note:** * RCOPE (Religious + Coping) = a validated scale that measures positive and negative ways of using religion to cope with stress.

## Data Availability

No new data were created or analyzed in this study.
